# High expression of CTHRC1 promotes EMT of epithelial ovarian cancer (EOC) and is associated with poor prognosis

**DOI:** 10.18632/oncotarget.5358

**Published:** 2015-10-03

**Authors:** Minzhi Hou, Zhiqiang Cheng, Hongwei Shen, Shanyang He, Yang Li, Yunping Pan, Chongjin Feng, Xinlin Chen, Yang Zhang, Millicent Lin, Liantang Wang, Zunfu Ke

**Affiliations:** ^1^ Department of Pathology, the First Affiliated Hospital, Sun Yat-sen University, Guangzhou, Province Guangdong, P.R. China; ^2^ Department of Pathology, ShenZhen People's Hospital, Second Clinical Medical College of Jinan University, Shenzhen, Guangdong, P.R. China; ^3^ Department of Gynecology, the First Affiliated Hospital, Sun Yat-sen University, Guangzhou, Province Guangdong, P.R. China; ^4^ Department of Stomatology, the First Affiliated Hospital, Sun Yat-sen University, Guangzhou, Province Guangdong, P.R. China; ^5^ Department of Preventive Medicine and Biostatistics, School of Basic Medical Science, Guangzhou University of Chinese Medicine, Guangzhou, P.R. China; ^6^ Biomedical Engineering, University of Texas at El Paso, El Paso, Texas, USA; ^7^ Department of Molecular and Medical Pharmacology, Crump Institute for Molecular Imaging (CIMI), California NanoSystems Institute (CNSI), University of California, Los Angeles, California, USA

**Keywords:** CTHRC1, epithelial-mesenchymal transition, epithelial ovarian cancer, β-catenin

## Abstract

Collagen triple helix repeat-containing 1 (CTHRC1) is aberrantly overexpressed in multiple malignant tumors. However, the expression characteristics and function of CTHRC1 in epithelial ovarian cancer (EOC) remain unclear. We found that CTHRC1 expression was up-regulated in the paraffin-embedded EOC tissues compared to borderline or benign tumor tissues. CTHRC1 expression was positively correlated with tumor size (*p* = 0.008), menopause (*p* = 0.037), clinical stage (*p* = 0.002) and lymph node metastasis (*p* < 0.001) and was also an important prognostic factor for the overall survival of EOC patients, as revealed by Kaplan-Meier analysis. CTHRC1 increased the invasive capabilities of EOC cells *in vitro* by activating the Wnt/β-catenin signaling pathway. We showed that ectopic transfection of CTHRC1 in EOC cells up-regulated the expression of EMT markers such as N-cadherin and vimentin, and EMT-associated transcriptional factor Snail. Knockdown of CTHRC1 expression in EOC cells resulted in down-regulation of N-cadherin, vimentin, Snail and translocation of β-catenin. Collectively, CTHRC1 may promote EOC metastasis through the induction of EMT process and serve as a potential biomarker for prognosis as well as a target for therapy.

## INTRODUCTION

Epithelial Ovarian Cancer (EOC) accounts for more than 80% of ovarian cancer. Though the incidence of EOC is extremely low, it is the leading cause of mortality among gynecologic malignancies globally, and its incidence has been increasing persistently in developing countries [1, 2]. Despite the rapid development of surgical and chemotherapy technologies, treatment of advanced stage EOC still remains a challenge [3, 4]. According to GLOBCAN 2008, over 225,500 estimated new cases and 140,200 estimated deaths of EOC occurred worldwide in 2008 [2]. Because of nonspecific symptoms and lack of effective methods for detection at earlier stages, only 20% of EOCs are eventually confirmed in stage I, while the majority of EOC patients are diagnosed at advanced stages (III and IV) [3, 4]. Therefore, it is of great importance to develop early detection methods for ovarian cancer to improve the survival rate. Identification of specific and sensitive biomarkers for the early diagnosis of EOC may pave the way for prolonging the survival time of EOC.

Epithelial-Mesenchymal Transition (EMT), which is characterized by the loss of epithelial markers and the acquisition of mesenchymal markers, as well as a fundamental change in cellular morphology and phenotype with increased ability to migrate, is considered an important step in metastasis [5]. Numerous signaling pathways, such as RTKs, Wnt, TGF-β, and NF-κB, mediate the EMT process by activating a series of master transcriptional regulators of the Slug, Snail, Twist and ZEB1 families [5]. Thus, dissecting the molecular mechanisms regulating the EMT process is pivotal in the understanding of tumor invasiveness and metastasis. The Wnt/β-catenin pathway is reported to be involved in EMT by decreasing the cell-cell contact protein E-cadherin to promote the migration and invasion of cancer cells [5, 6]. However, exactly how the Wnt/β-catenin pathway is aberrantly activated in EOC remains unknown.

Collagen triple helix repeat containing-1 (CTHRC1) is a 28-kD extracellular matrix glycoprotein that shares 92% of homolog sequences in human compared to that of rat [7, 8]. Aberrant CTHRC1 expression is detected in several malignant tumors, including melanoma, and cancers of the gastrointestinal tract, esophagus, breast, ovary, thyroid, liver and pancreas [7, 9, 10]. As a key downstream target of the DPAGT1/canonical Wnt pathway feedback loop, CTHRC1 activates the non-canonical Wnt pathway to promote oral squamous cell carcinoma (OSCC) cell migration [11]. Furthermore, being a prognostic factor, CTHRC1 promotes the invasiveness of gastrointestinal stromal tumors by activating Wnt/PCP-Rho signaling. Ke *et al*. reported that CTHRC1 mediates non-small cell lung cancer (NSCLC) aggressiveness via the GSK-3β/β-catenin pathway [12]. Currently, there is little information about the clinical significance of CTHRC1 in EOC and the molecular mechanisms by which CTHRC1 promotes the invasion and metastasis of EOC.

Advances in high-throughput technologies have aroused great interest in discovering tumor-biomarkers for ovarian cancer by methods like targeted sequencing or proteomics [13]. Proteomic techniques can be used to identify the molecular changes during the process of disease. In our study, we first used comparative proteomic analysis to verify the aberrant expression of CTHRC1 in ovarian cancer tissues compared with benign ovarian tissues. CTHRC1 overexpression induced ovarian cancer cell lines to undergo EMT and promoted cell migration and invasion through activating Wnt/β-catenin signaling, making CTHRC1 a potential target for EOC treatment.

## RESULTS

### Differential CTHRC1 expression in EOCs and control tissues

**Figure 1:**
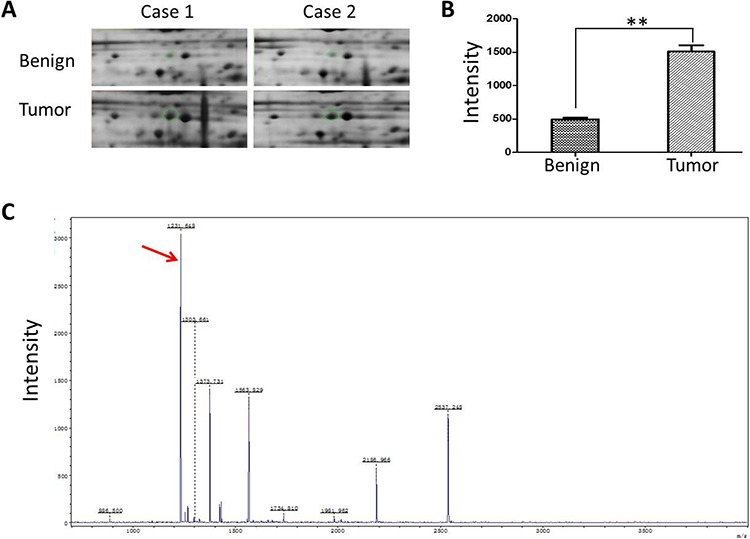
Identification of CTHRC1 high expression in EOC **A.** A representatively enlarged image of two-dimensional gels with CTHRC1 up-regulation in EOC tissues compared to the corresponding adjacent ovarian tissues. The area in the green loop represents the expression density of CTHRC1 protein. **B.** The densitometric analysis for CTHRC1 from 8 patients was semi-quantitatively calculated using PDQuest software. Each bar represents the mean ± SEM of intensity (**p < 0.01). **C.** MS identification of in-gel trypsin digests of the target protein and analysis of the depicted peptide spectrum resulted in the identification of CTHRC1. Red arrow marks the special peak of CTHRC1 protein.

To identify the differential protein expression spectrum between EOCs and their corresponding adjacent benign ovarian tissues, we extracted protein from 8 EOC and used comparative proteomic analysis. All the protein spots of interest were identified by MALDI-TOF MS analysis and were further confirmed by a comparative sequence search in the Mascot database. Figure 1 exhibited the peptide mass fingerprinting (PMF) of representative CTHRC1.

### Validation of CTHRC1 expression in EOC tissues and control tissues

**Figure 2:**
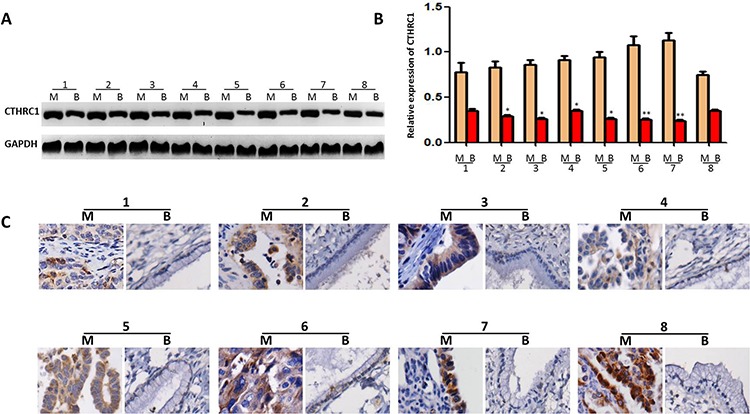
CTHRC1 expression in EOC tissues and adjacent ovarian benign tumor samples **A.** CTHRC1 expression was tested by Western blotting in each of the primary EOC tissue (M) and adjacent benign mucinous or serous epithelium tissues (B) from the same patient. **B.** CTHRC1 relative expression was normalized for GAPDH. Error bars represent mean ± SEM from three independent experiments (*p < 0.05, **p < 0.01). **C.** Elevated expression of CTHRC1 in protein levels was further confirmed by IHC.

We used Western blots to examine CTHRC1 expression in tissue samples used for proteomic analysis (EOC tissues, *n* = 8 and benign ovarian tumor tissues, *n* = 8). Despite intragroup variation of CTHRC1, our results showed that CTHRC1 protein expression was higher in all eight pairs of fresh EOC tissues compared to that of their corresponding adjacent ovarian tissues (Fig. 2A, 2B). This was further confirmed by immunohistochemical (IHC) results (Fig. 2C). These data indicate that CTHRC1 expression is up-regulated in EOC patients.

### The relationship between CTHRC1 expression and EOC clinicopathological parameters

**Figure 3:**
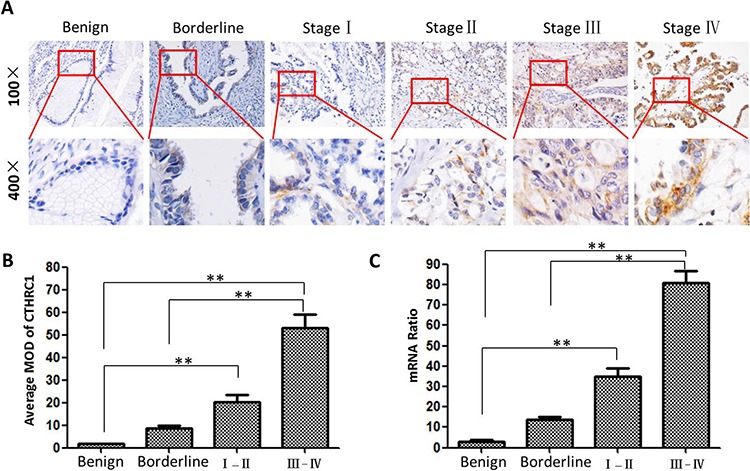
CTHRC1 protein and mRNA expression in EOC patients with different clinical stages **A.** Representative IHC images of CTHRC1 expression in benign (*n* = 22), borderline (*n* = 17) and malignant condition (I-II: *n* = 28 and III-IV :*n* = 60), respectively. **B.** Statistical analyses of the average MOD for CTHRC1 staining in different disease status (** *p* < 0.01). **C.** CTHRC1 mRNA levels were analyzed by real time RT-PCR and expressed as CTHRC1/β-actin mRNA ratio (**p < 0.01).

Paraffin-embedded tissues (88 EOC tissues, 17 ovarian borderline tissues and 22 ovarian benign epithelial tissues) were selected to examine the clinical significance of CTHRC1 in EOCs by IHC. Table 1 shows that CTHRC1 expression is correlated with tumor size (*p* = 0.008), metastasis status (*p* = 0.037), clinical stage (*p* = 0.002), and lymph node metastasis (*p* < 0.001). However, there was no association of CTHRC1 expression with age, tumor grade and tumor subtype. Furthermore, CTHRC1 expression was stronger in EOC tissues than that in the borderline and benign tissues (*p* = 0.025). CTHRC1 staining intensity gradually increased in accordance with malignancy: from benign, borderline, early stage to advanced stage (*p* < 0.001, Fig. 3A and 3B). This was further verified at the mRNA level by real time PCR (Fig 3C).

**Table 1:** Oligonucleotide primer sequences used in this study

Gene	Forward (5′-3′)	Reverse (5′-3′)
CTHRC1	TGGACACCCAACTACAAGCA	GAACAAGTGCCAACCCAGAT
CD133	GGGAGAACAATAATAGGATATTTTGAA	CGATGCCACTTTCTCACTGAT
CD44	CCCTGCTACCAGAGACCAAGAC	GCAGGTTCCTTGTCTCATCAGC
N-cadherin	ACAGTGGCCACCTACAAAGG	CCGAGATGGGGTTGATAATG
Vimentin	GAGAACTTTGCCGTTGAAGC	GCTTCCTGTAGGTGGCAATC
E-cadherin	TGCCCAGAAAATGAAAAAGG	GTGTATGTGGCAATGCGTTC
Snail	CAGACCCACTCAGATGTCAA	CATAGTTAGTCACACCTCGT
Slug	GGTCAAGAAGCATTTCAAC	GGTAATGTGTGGGTCCGA
Twist	GGGAGTCCGCAGTCTTAC	CCTGTCTCGCTTTCTCTTT
β-actin	GCATGGGTCAGAAGGATTCCT	TCGTCCCAGTTGGTGACGAT

### A prognostic role for CTHRC1 expression in EOC patients

**Figure 4:**
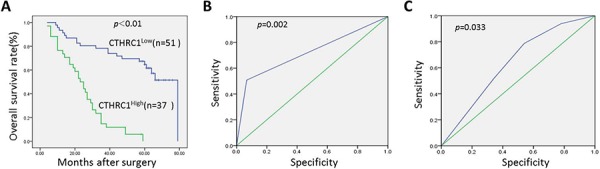
Kaplan-Meier survival curves according to CTHRC1 status and its corresponding Receiver Operating Characteristic analysis **A.** Prognostic significance assessed by Kaplan-Meier analysis and log-rank tests. Overexpression of CTHRC1 predicts lower OS rate. **B.** The cutoff of CTHRC1 has optical sensitivity and specificity for metastasis status (area under the curve was 0.746, 95% CI: 0.629–0.863; *p* = 0.002). **C.** The cutoff of CTHRC1 has optical sensitivity and specificity for EOC recurrence (area under the curve was 0.638, 95% CI: 0.519–0.758; *p* = 0.033).

Previous studies report CTHRC1 overexpression as a poor survival factor in many cancers [12, 14, 15], but its prognostic role in EOC is unknown. In this study, Kaplan-Meier analysis and log-rank test showed that high CTHRC1 expression in EOCs predicted poor survival. Median overall survival in the high CTHRC1 expression subgroup was 25.0 months (95% CI, 19.224–30.776), while in the follow-up interval, the low CTHRC1 expression subgroup had a cumulative survival rate of approximately 0.58 (Fig. 4A). As shown in Fig. 4B and 4C, CTHRC1 levels predicted EOC metastasis and recurrence. The areas under the curves are 0.638 (95% CI: 0.519–0.758; *p* = 0.033) and 0.746 (95% CI: 0.629–0.863; *p* = 0.002), respectively.

In the univariate analysis, clinical stage, tumor grade and CTHRC1 levels were correlated with disease free survival of EOC patients (*p* = 0.001, 0.015 and < 0.001, respectively; Table 2). Age, menopause, clinical stage, tumor grade, tumor type, tumor size, lymph node metastasis and CTHRC1 level were correlated with the overall survival (*p* = 0.039, 0.011, < 0.001, 0.001, 0.025, 0.014, < 0.001 and 0.004, respectively; Table 2). To determine whether CTHRC1 expression level was an independent predictor for EOC patients’ recurrence and survival time, a multivariate analysis was performed using COX proportional hazard regression model, together with age, clinical stage and other clinical parameters. Again, CTHRC1 expression was positively correlated with EOC patients’ recurrence and survival time (*p* < 0.001 and = 0.003, respectively; Table 3 and 4).

**Table 2:** Correlation between CTHRC1 expression and clinicopathologic characteristics of EOC

Characteristics	CTHRC1 expression		Chi-square test	
	Low (0–2+)		High (3+)	*p*-value
Benign tissues	22	22	0	0.025
Borderline tissues	17	16	1	
EOC tissues	88	51	37	
Age(years)				0.071
>50	47	23	24	
≤50	41	28	13	
Tumor size				0.008
>1 cm	17	5	12	
≤1 cm	71	46	25	
Menopause				0.037
Yes	48	23	25	
No	40	28	12	
Clinical stage				0.002
I-II	28	23	5	
III-IV	60	28	32	
Tumor grade				0.363
High	22	15	7	
Medium	39	23	16	
Low	27	13	14	
Tumor subtype				0.302
Serous	63	33	30	0.194*
Mucinous	14	10	4	
Endometrioid	4	2	2	
Clear cell	1	1	0	
Others	6	5	1	
Lymph node metastasis				<0.001
No	61	38	23	
Yes	24	10	14	

* *p* = 0.194: serous vs mucinous.

**Table 3:** Univariate survival analysis in EOC patients

	Overall survival		Disease-free survival	
	HR (95%CI)	*p* value	HR (95%CI)	*p* value
Age (years)(>50 vs. 50)	1.80 (1.03, 3.16)	0.039	1.37 (0.70, 2.29)	0.362
Menopause (no vs. yes)	0.48 (0.27, 0.85)	0.011	0.75 (0.38, 1.48)	0.751
Stage (III/IV vs. I/II)	7.50 (2.96, 19.01)	<0.001	6.45 (2.25, 18.46)	0.001
Tumor grade (II-III vs. I)	4.40 (1.82, 10.61)	0.001	3.39 (1.27, 9.04)	0.015
Tumor type (others vs. serous+mucinous)	0.20 (0.05, 0.82)	0.025	0.29 (0.07, 1.21)	0.088
Lymph node metastasis (yes vs. no)	1.72 (1.18, 2.65)	0.014	1.27 (0.71, 2.29)	0.426
Tumor size (>1 cm vs. ≤1 cm)	2.51 (1.34, 4.70)	0.004	1.98 (0.88, 4.43)	0.097
CTHRC1 expression (high vs. low)	4.55 (2.54, 8.15)	<0.001	3.94 (1.96, 7.95)	< 0.001

**Table 4:** Multivariate analysis of overall survival in EOC patients

	B	Hazard ratio	95.0% CI for Exp(B)		*p*-value
			Lower	Upper	
Stage (III/IV vs. I/II)	1.465	4.33	1.63	11.49	0.003
Tumor grade (II-III vs. I)	1.434	4.20	1.63	10.80	0.003
Lymph node involvement(yes vs. no)	0.691	2.00	1.19	3.35	0.009
CTHRC1 expression (high vs. low)	1.231	3.42	1.82	6.43	< 0.001

### Effects of CTHRC1 expression on the malignant phenotype of EOC cells

**Figure 5:**
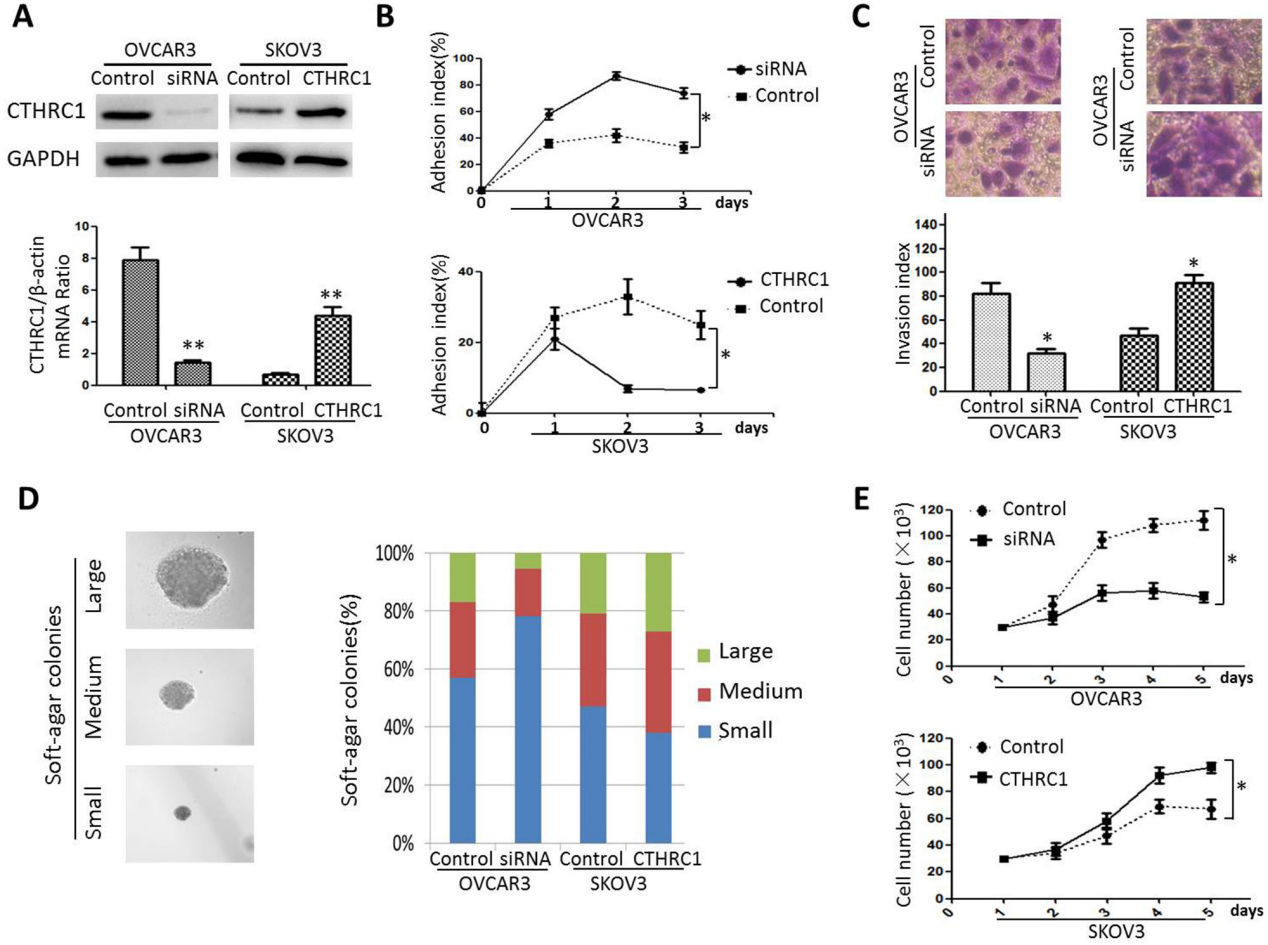
Effect of CTHRC1 gain/loss of function on EOC cell malignant phenotype **A.** Down-regulation and up-regulation of CTHRC1 by CTHRC1-siRNA and pcDNA3.1-CTHRC1, respectively. Data are means ± SD of 3 independent experiments (**p < 0.01). **B.** 48 h after transfection, cells were seeded in the null-adhesion condition (day 0). At days 2–3, an adhesion index (%) was calculated as [n cells grown in adhesion/(n cells grown in adhesion + n cells grown in suspension) × 100%]. CTHRC1 significantly inhibited EOC cell adhesion (*p < 0.05). **C.** Transwell assay showed that cell invasiveness was diminished by CTHRC1-siRNA treatment, and enhanced by pcDNA3.1-CTHRC1 treatment (*p < 0.05). **D.** Transfected EOC cells were also tested for the ability to form colonies in soft agar: 7 days after seeding in soft agar, transfected cell samples were analyzed by light microscopy and the size/number of colonies was evaluated, considering percentage fractions. **E.** Cell viability was tested by MTT assay: cell viability was impaired by CTHRC1-siRNA treatment (the upper), and elevated by pcDNA3.1-CTHRC1 treatment (the lower). Data are from three separate experiments (*p < 0.05).

To further investigate the correlation between CTHRC1 gain/loss and EOC cells’ malignant phenotypes, we performed a series of cell function assays. In order to overexpress CTHRC1, SKOV3 cells were transfected with a pcDNA3.1-CTHRC1 plasmid in which a full coding sequence of cDNA for the endogenous human CTHRC1 was cloned. Contrarily, CTHRC1 silencing was achieved in OVCAR3 cells by using a pcDNA3.1 plasmid expressing a siRNA against CTHRC1. An empty pcDNA3.1 plasmid was used as negative control (Fig. 5A). Seventy-two h after transfection (day 0), EOC cells were seeded in untreated culture dishes to impede cell adhesion. At days 1–3, an adhesion index (%) was calculated as [n cells grown in adhesion/(n cells grown in adhesion + n cells grown in suspension) × 100%]. CTHRC1 up-regulation led to a drastic decrease in the cell adhesion index, whereas the opposite effect was observed in EOC cells treated with CTHRC1 siRNA (Fig. 5B). Additionally, in Transwell assay, the invasiveness of EOC cells was positively correlated with CTHRC1 expression (high CTHRC1 level - high invasion index, and vice versa) (Fig. 5C). Furthermore, we assessed the capability of EOC cells to form colonies on soft agar. Interestingly, the growth of EOC cells were notably inhibited by CTHRC1-siRNA and promoted by pcDNA3.1-CTHRC1 (Fig. 5D). We also evaluated the effect of CTHRC1 up-regulation/down-regulation on cell proliferation. CTHRC1 up-regulation promoted the growth of SKOV3 cells. Conversely, CTHRC1 silencing inhibited the viability of OVCAR3 cells (Fig. 5E). Collectively, our data show that CTHRC1 inhibits epithelial cell adhesion and promotes malignant features such as survival advantage, proliferation and invasiveness of EOC cells.

### Wnt/β-catenin pathway activation contributes to CTHRC1-mediated EMT

**Figure 6:**
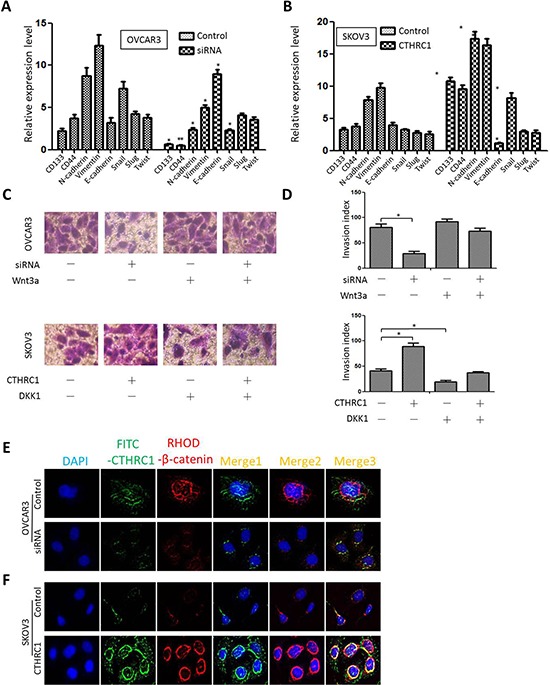
Effect of CTHRC1 on stem/EMT-related gene expression and Wnt/β-catenin signaling **A, B.** Relative mRNA expression of CD133, CD44, N-cadherin, vimentin, E-cadherin, Snail, Slug and Twist was evaluated by real time PCR in transfected OVCAR3 and SKOV3 cells, 72 h after transfection. Data were normalized against β-actin mRNA expression (*n* = 3) (*p < 0.05 and **p < 0.01). Data are from three separate experiments. **C, D.** The inhibiting effect of CTHRC1-siRNA on the invasiveness of EOC cells was reversed by Wnt3a factor. The promoting effect of pcDNA3.1-CTHRC1 on the invasiveness of EOC cell was blocked by DKK1. Quantification analysis of migrated cells were performed for six randomly selected fields (original magnification: 200 ×) (*p < 0.05). **E, F.** Immunofluorescent staining was performed to assess β-catenin protein sublocalization in OVCAR3 cells transfected with siRNA-CTHRC1 and SKOV3 cells treated with pcDNA3.1-CTHRC1, respectively. CTHRC1 overexpression promoted β-catenin nuclear localization.

The mRNA levels of cancer stem-related genes (CD133, CD44), EMT-related markers (N-cadherin, vimentin and E-cadherin) and transcriptional factors (Snail, Slug and Twist) were detected under different experimental conditions. SKOV3 cells transfected with pcDNA3.1-CTHRC1 showed low E-cadherin mRNA levels and higher mRNA levels of CD133, CD44, N-cadherin, vimentin and Snail, compared to the control. Conversely, OVCAR3 cells treated with CTHRC1-siRNA exhibited higher E-cadherin mRNA levels and lower mRNA levels of CD133, CD44, N-cadherin, vimentin and Snail than those in the control (Fig. 6A and 6B). We also found that the inhibiting effect of CTHRC1-siRNA on the invasiveness of OVCAR3 cells could be reversed by Wnt3a. Simultaneously, DKK1 might block the promoting effects of CTHRC1 on SKOV3 cell invasion (Fig. 6C and 6D). In addition, we demonstrated a strong relationship between Wnt/β-catenin pathway activation and CTHRC1 status. Overexpression of CTHRC1 led to Wnt/β-catenin pathway activation accompanied by the nuclear accumulation of β-catenin in the immunofluorescence assay. On the contrary, CTHRC1 silencing remarkably decreased β-catenin protein levels in the nucleus (Fig. 6E and 6F).

**Figure 7:**
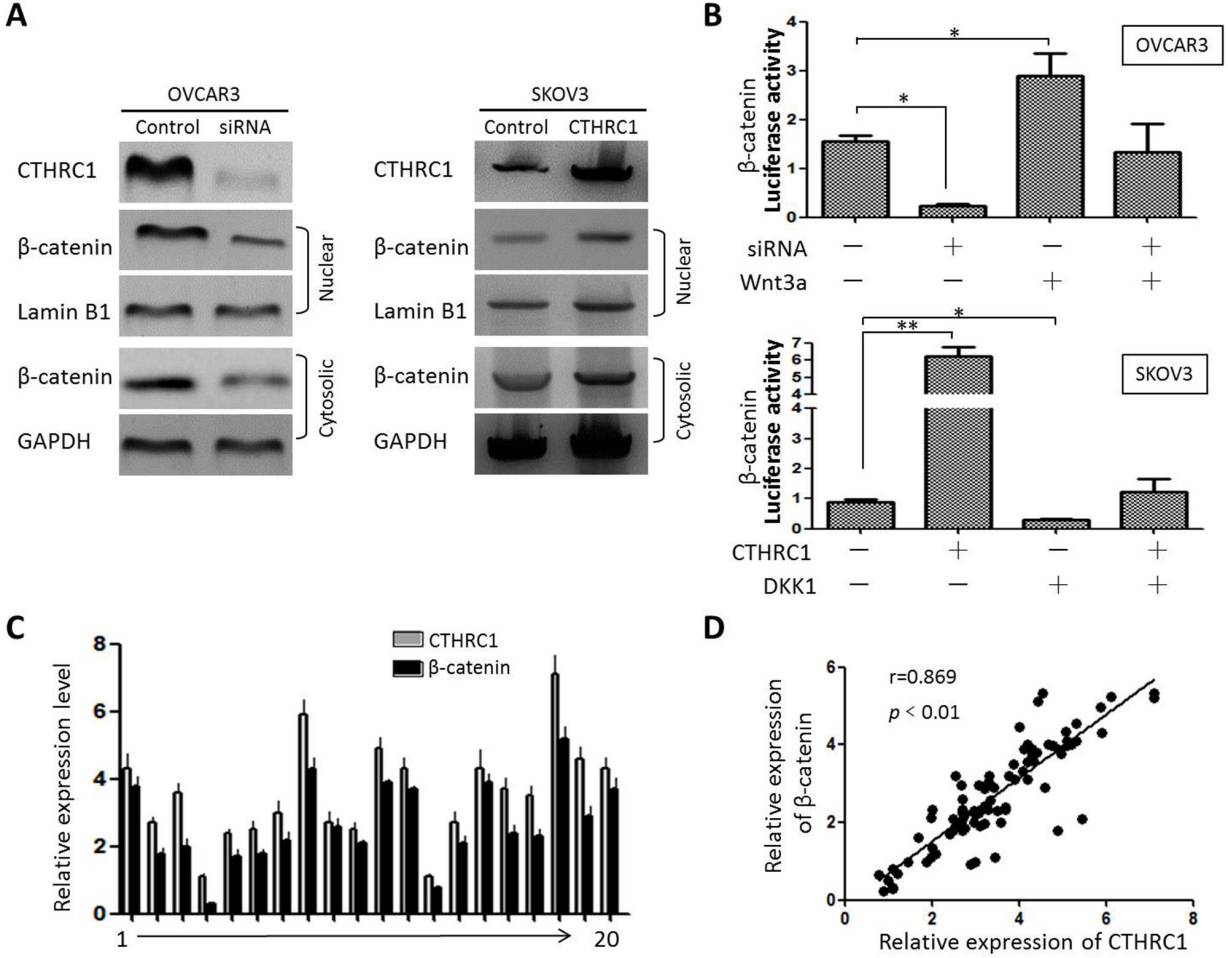
Relationship between CTHRC1 and β-catenin expression **A.** Western blot analysis of β-catenin in cytoplasm (GAPDH as control) and nucleus (Lamin B1 as control) of the cultured cells showed that down-regulation of CTHRC1 reduced nuclear β-catenin and CTHRC1 overexpression resulted in increased nuclear β-catenin. **B.** Luciferase reporter assay demonstrated relatively decreased luciferase activity of β-catenin in OVCAR3 cells after being transfected with CTHRC1-siRNA, and was reversed by ectogenic Wnt3a. CTHRC1 overexpression increased the luciferase activity of β-catenin in SKOV3 cells, which was significantly blocked by the Wnt/β-catenin inhibitor such as DKK1. Data are from three separate experiments (*p < 0.05 and **p < 0.01). **C, D.** Relative protein expression of CTHRC1 and nuclear β-catenin in 88 EOC tissue samples was tested by Western blotting. Representative expression spectrum for 20 patients was provided. CTHRC1 and nuclear β-catenin expression was normalized against GAPDH protein. The Pearson correlation coefficient was calculated (*r* = 0.869) (*p* < 0.01). Data are from three separate experiments.

We further evaluated the effect of CTHRC1 on the subcellular localization of β-catenin. Interestingly, CTHRC1 down-regulation led to a decrease of β-catenin in the nucleus (Fig. 7A). Moreover, luciferase reporter assays demonstrated that the activity of β-catenin was enhanced by CTHRC1 up-regulation in SKOV-3 cells, which could be weakened by DKK1. In contrast, Wnt3a could recover the inhibitory effects of CTHRC1-siRNA on the activity of β-catenin in OVCAR3 cells (Fig. 7B). To further verify the correlation between CTHRC1 and β-catenin, we analyzed 88 EOC clinical samples by Western blot. Our results show that CTHRC1 expression is positively correlated with nuclearβ-catenin protein levels (*r* = 0.869, Fig. 7C and 7D and Supplementary Table 1).

### CTHRC1 is closely associated with EMT status in xenograft models

**Figure 8:**
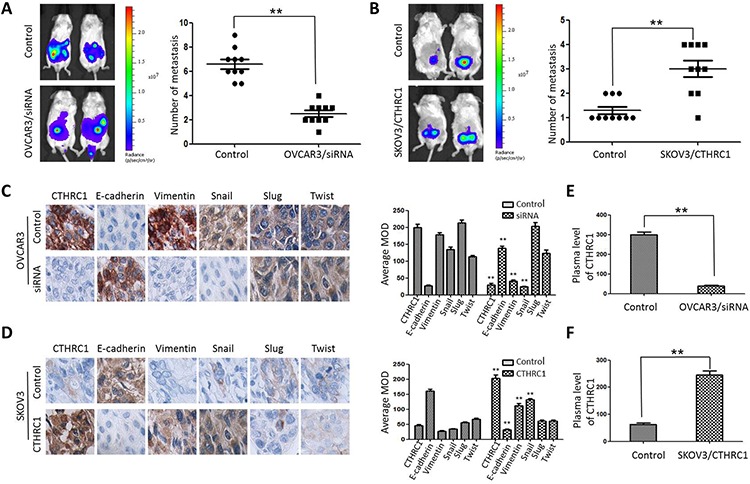
CTHRC1 promoted tumor metastasis by regulating EMT *in vivo* **A, B.** Representative BLI images of mice bearing OVCAR3/CTHRC1-siRNA or SKOV3/pcDNA3.1-CTHRC1 tumors with metastatic lesions. Mice (*n* = 10) were imaged six weeks later to observe local tumor growth and metastasis (**p < 0.01). **C, D.** CTHRC1 promoted the EMT process *in vivo*. IHC demonstrated that CTHRC1 increased vimentin and Snail expression, and decreased E-cadherin expression in the primary xenograft tumor tissues (magnification: 200 ×). **E, F.** In the mice with OVCAR3/CTHRC1-siRNA xenograft tumor, the CTHRC1 protein levels in the plasma were lower than that in the control. In contrast, in the SKOV3/pcDNA3.1-CTHRC1 group, the CTHRC1 protein levels in the plasma were higher than that in the control (**p < 0.01).

To reveal the role of CTHRC1 in the metastasis and EMT of EOC cells, we explored the metastatic activity of OVCAR3/CTHRC1-siRNA and SKOV3/pcDNA3.1-CTHRC1 in nude mice. We found that OVCAR3/CTHRC1-siRNA cells predominantly localized to tumor nodules in the primary injection sites compared to control. However, SKOV3/pcDNA3.1-CTHRC1 cells formed multiple tumors in the peritoneum cavity. The number of metastatic nodules was measured according to the fluorescence signal and H&E staining. As shown in Fig. 8A and 8B, SKOV3/pcDNA3.1-CTHRC1 cells formed a greater number of metastases than SKOV3 cells in abdomen (3.00 ± 1.05 vs. 1.30 ± 0.48, *p* < 0.01, respectively). These *in vivo* results confirmed the role of CTHRC1 in the promotion of EOC invasion.

EMT may aberrantly take place in epithelial neoplasms whereby cancer cells lose their epithelial characteristics, such as cell-to-cell contacts, and are endowed the ability to metastasize during cancer progression. Our IHC results show that the majority of tumor cells in SKOV3/pcDNA3.1-CTHRC1 xenografts expressed a more intense vimentin signal, but exhibited weaker E-cadherin staining, two key markers for tumor metastasis, indicating that these cells were undergoing the EMT process (Fig. 8C and 8D). Moreover, these cells also exhibited high levels of Snail, an important EMT-associated transcriptional factor. As shown in Fig. 8E and 8F, the serum levels of CTHRC1 in the SKOV3/pcDNA3.1-CTHRC1 xenografts were much higher than that in the SKOV3 controls (**p < 0.01).

## DISCUSSION

Recent molecular biological characterizations describe EOC as a very heterogeneous disease, having a variable degree of malignancy ranging from curable to a strongly malignant carcinoma [16]. Despite enormous efforts to develop effective anti-cancer drugs [17–25], there has been little improvement in the poor outcome of EOC over the past decades due to a high recurrence rate and the tendency to disseminate within the peritoneal cavity. Therefore, developing novel predictive biomarkers for recurrence and metastasis is urgently needed to improve the survival of patients with EOC.

One important reason for poor EOC treatment is that there are many molecules involved in promoting cancer invasion and metastasis [26]. Therefore, elucidating the exact functions of these molecular targets may improve the outcomes of EOC patients. Gene expression profiling analysis from Vladimir Lazar *et al*. identified CTHRC1 as a potential biomarker for early diagnosis of NSCLC [27]. In our study, by analyzing differential protein expression between EOCs and benign ovarian tissues, an increase of CTHRC1 expression was identified during the genesis of EOC. CTHRC1 was originally identified as an overexpressed gene in balloon-injured arteries, associating with myofibroblasts and sites of collagen matrix deposition [28]. Aside from functioning in the context of injured arteries, CTHRC1 has been demonstrated to act as a positive regulator of osteoblastic bone formation to increase bone mass [29]. Recently, experiments have shown that CTHRC1 is widely present in human solid tumors and seems to be associated with invasion and metastasis [7, 30]. Similarly, our Western-blot and immunohistochemical results confirmed the proteomic data that there was a significant increase in CTHRC1 protein expression in EOC tissues, compared to adjacent benign ovarian tissues, suggesting its promoting functions in EOC development. Furthermore, CTHRC1 expression gradually increased in accordance with malignant degrees from benign, borderline, early stage to advanced stage, which was further confirmed at the mRNA level. Taken together, these data suggest that CTHRC1 aberrant overexpression contributes to EOC carcinogenesis and progression.

By analyzing the expression characteristics of CTHRC1 in the clinical EOC samples, we found that CTHRC1 levels in EOCs with lymph node metastasis were remarkably higher than those in EOCs without lymph node metastasis. These results strongly indicate that CTHRC1 may promote EOC metastasis. The classification of EOC staging, revised in 2014 by the FIGO (International Federation of Gynecologists and Obstetricians), was widely accepted as the standard to predict the prognosis of EOC patients [31]. Additionally, valid prognostic factors in EOC patients include the presence and extent of residual tumor after cytoreductive surgery, histology, age, performance status, and differentiation status [32]. CTHRC1 was documented as a prognostic predictor in many cancers such as Gastrointestinal Stromal Tumors (GIST), non-small cell lung cancer (NSCLC), gastric cancer, and colorectal cancer [12, 14, 33–35]. We have assessed the correlation between CTHRC1 expression and EOC clinicopathological parameters and revealed that CTHRC1 levels were closely correlated with tumor size and clinical stage. Moreover, Kaplan-Meier analysis revealed that EOC patients with high CTHRC1 expression have significantly shorter OS. Both univariate and multivariate analyses also suggest that CTHRC1 expression is an independent prognostic factor for OS and DFS in EOC patients. In addition, there is high clinical value for CTHRC1 in predicting metastasis and risk of recurrence in postoperative EOC patients as well as contributing to the improvement of the clinical therapeutic effects. These analyses suggest that CTHRC1-positive EOCs exhibit a greater likelihood of aggressive and malignant behavior.

Previous studies reported a significant difference in high CTHRC1 expression between male and female GIST patients [14], suggesting a relationship between male hormones (e.g. androgen and testosterone) and CTHRC1 expression. Furthermore, postmenopausal ovaries continue to secrete a large amount of testosterone and a moderate amount of androstenedione [36]. Interestingly, our data also demonstrated that CTHRC1 protein levels were much higher in postmenopausal women than in premenopausal. However, the exact interaction and their corresponding clinical significance between CTHRC1 and sex hormones required in-depth investigation.

To date, how CTHRC1 promotes cancer cell migration and adhesion remains unclear. We explored the molecular mechanism through which CTHRC1 activates the migration of EOC cells by investigating its effects on the EMT. The ability of cancer cells to invade into local tissues is influenced by EMT. A number of distinct molecular processes are engaged in EMT, including the expression profiling of specific cell-surface proteins [37]. E-cadherin is an epithelial calcium-binding transmembrane glycoprotein, whose down-regulation is the prototypical biomarker of EMT and enables tumor cells to acquire an invasive phenotype [38, 39]. CTHRC1 overexpression led to significant decrease in the levels of E-cadherin. Furthermore, we found that treatment with CTHRC1-siRNA diminished the migration of EOC cells induced by pcDNA3.1-CTHRC1 treatment, suggesting that autocrine and paracrine activation of the CTHRC1 pathway is critical for cell motility in EOC cells. Aberrant expression of N-cadherin in the selected cell lines promotes invasion and metastasis [40]. Expression of cytoplasmic N-Cadherin correlates significantly with poor histological differentiation, enhanced motility and TGF-β-1 activation [41, 42]. Simultaneously, up-regulated mesenchymal marker vimentin expression was associated with highly invasive and metastatic properties along with the expression of cancer stem cell (CSC) markers [43]. Our *in vitro* cell models, EOC cells transfected with pcDNA3.1-CTHRC1, displayed a dramatic increase of N-cadherin and vimentin. These cells clearly exhibited mesenchymal characteristics, such as the acquisition of mesenchymal phenotype concomitant with CD133/CD44 high expression pattern and elevated mammosphere-forming ability. Consistently, CTHRC1 also promoted the growth capacity of EOC cells. These results strongly suggest that CTHRC1 induces EMT process.

Although the functional roles of CTHRC1 in tumor cell EMT are well established, the underlying mechanisms of how CTHRC1 regulates the EMT process are unclear. Recent findings revealed that CTHRC1 stabilizes the Wnt-Fzd complex [44]. Binding of Wnts to Frizzled receptors leads to accumulation and nuclear translocation of β-catenin, forming a complex with TCF4 that drives the transcription of metastasis-related genes [45]. Moreover, in most conditions, a group of transcription factors downstream of Wnt/β-catenin signaling play a significant role in the regulation of EMT and cancer metastasis [46–48]. In addition, accumulation of stabilized β-catenin, especially nuclear β-catenin, is an important marker of activated Wnt signaling [49]. We showed that CTHRC1 promotes EMT by activating the Wnt/β-catenin pathway, which is supported by a significant increase in Wnt/β-catenin transcriptional activity and an accumulation of nuclear β-catenin. Moreover, we found that treatment with Dkk1, a specific inhibitor of Wnt/β-catenin signaling [50], diminished the migration ability and activity of Wnt/β-catenin signaling in EOC cells induced by CTHRC1 overexpression. This suggests that CTHRC1-induced activation of Wnt/β-catenin signaling accounts for its effect on cell proliferation and motility. However, exactly how CTHRC1 activates the Wnt/β-catenin pathway needs to be further explored.

In conclusion, the data we collected delineates the function and mechanism of CTHRC1 in EMT and metastasis of EOC. We show that CTHRC1 underlies the onset of EMT and aggressive metastasis of EOC by activating the Wnt/β-catenin signaling. We have demonstrated that high levels of CTHRC1 are closely correlated with clinical stage of FIGO classification and prognosis of EOC, suggesting that CTHRC1 serves as a candidate cancer biomarker for the recurrence risk and prognosis in post-operative EOC patients.

## MATERIALS AND METHODS

### Cell lines

Human ovarian carcinoma cell lines SKOVR3 and OVCAR3 were donated by the gynecological lab of the Affiliated Third Hospital, Sun Yat-sen University. SKOV3 and OVCAR3 cells were maintained in RPMI 1640 (GibCo BRL, Grand Island, NY, USA) supplemented with 10% fetal bovine serum (GibCo BRL, Grand Island, NY, USA), and 100 U/mL penicillin-streptomycin mixture (GibCo BRL, Grand Island, NY, USA) at 37°C in a humidified atmosphere of 5% CO2. CTHRC1 overexpression plasmid pcDNA3.1-CTHRC1 and CTHRC1 siRNA (RiboBio, China) were transiently transfected using Lipofectamine 2000 (Invitrogen, USA). The concentration of DKK1 (R&D, USA) in the medium was 15 ng/mL. Wnt3a-conditioned medium (Wnt3a-CM) was from L cells transfected with pGKWnt3a.

### Tissue specimen selection

All cases (*n* = 88) from January 1st, 2006 to December 31st, 2009 were included in this study, and they were collected according to the following criteria: 1). First-time diagnosed EOC patients without any pre-operation chemotherapy, radiotherapy and hormonal therapy; 2). Only found to possess gynecological tumor(s); 3). Subsequent chemotherapy followed by the cytoreductive surgery. Ovarian benign tumors (*n* = 22) and ovarian borderline tumors (*n* = 17) were selected as controls. The formalin-fixed, paraffin-embedded tissues of 88 malignant cases, 22 benign ovarian tumors and 17 borderline tumors were extracted from the surgical pathology archives of the Affiliated First Hospital, Sun Yat-sen University. The age of these selected patients at the time of first diagnosis ranged from 21 to 86 years (mean, 50. 9 years). Follow-up data were conducted using hospital medical records and telephone interviews. According to the criteria of the 2009 FIGO, 28 cases were evaluated in stage I-II and 60 were diagnosed as III-IV, and 24 were found lymph node(s) involvement, respectively. Tumors were graded according to the Silverberg grading system: 22 high-grade, 39 medium-grade and 27 low-grade. Our present study was approved by the medical ethics committee of Sun Yat-sen University.

### Immunohistochemical staining

Formalin-fixed, paraffin-embedded tissues sections (4 μm) from patients and mouse xenografts were made by rotary microtome (Leica, Wetzlar, Germany). All the slices were labeled with anti-CTHRC1 in 1:100 dilution (rabbit polyclonal antibody; Abcam, USA). 3,3-Diaminobenzidine tetrahydrochloride was used to visualize the staining reaction, and Mayer hematoxylin was counterstained subsequently. The number of positive-staining cells was estimated in a semi-quantitative method. Firstly, score of the staining intensity: colorless (0), buff (1), brownish yellow (2), and dark brown (3). Secondly, score of the percentage of positive cells: no positive cells (0), 10% positive cells or less (1), 11% to 50% positive cells (2), 51%to 75% positive cells (3), and more than 75% positive cells (4). The staining index was evaluated by multiplying the staining intensity score to the positive tumor cell score. Based on a measure of heterogeneity, a staining index of 3 or greater was defined as high expression, and 2 or lower as low expression. To monitor batch consistency of all the staining slices, we used the known positive control (human skin melanoma) and the negative control (an antibody dilution solution to take the place of primary antibody). The staining index was calculated using the Aperio ImageScope software (Aperio Technologies). We applied the mean optical density (MOD) to evaluate the staining intensity of each slide for the consistencies in IHC staining intensities. The MOD of each slices were determined by randomly selecting 5 representative fields, which were used to represent the whole tissue concentration of the stain or proportion of positive pixels.

### Proteomic analysis

We performed a proteomic analysis according to the previously described protocol [15], which included two-dimensional gel electrophoresis, gel visualization and assessment. Benign ovary tissue (*n* = 8) and EOC tissue (*n* = 8) samples were used to extract proteins for analysis. Tissue samples (~100 mg) were homogenised mechanically in lysis buffer (2 M thiourea, 7 M urea, 30 mM Tris, 4% CHAPS, 65 mM DTT and 2% Pharmalyte (pH 3–10; GE Healthcare, Piscataway, NJ, USA) by sonication on ice. The homogenates were separated by centrifugation at 15 000 r.p.m. for 1 h at 4°C. The supernatant was collected and stored in −80°C. 2-D cleanQ2 up kit (Amersham Biosciences, UK) and a 2-D Quant Kit (GE Healthcare, London, UK) were used to purify protein samples and qualify the concentration sequentially according to the manufacturers’ instructions. Proteins differentially expressed in two groups were identified by using two-dimensional gel electrophoresis and mass spectrometry. Two-dimensional gel electrophoresis was performed using an immobilized pH gradient (IPG) strip (24 cm, pH 3–10 NL; GE Healthcare), in which proteins were separated according to charge and molecular weight. Visualized stained-proteins were selected by using an Ettan Spot Handling Workstation (GE Healthcare), and the protein of interest were digested with trypsin. Then we performed the peptide mass-mapping using an ABI Voyager DE-STR mass spectrometer by matrix-assisted laser desorption time-of-flight mass spectrometry (MALDI-TOF MS). Furthermore, the masses of the tryptic peptides were used to search in the MASCOT Database (http://www.matrixscience.com/search_form_select.html) to identify the original protein. Search criteria of following were used: Homo sapiens, trypsin cleavage, with no constraints on either the molecular weight or the isoelectric point of the protein.

### Total RNA extraction and real-time RT-PCR

Total RNA was isolated from tissue specimens and cell lines using the RNAeasy kit (Qiagen, USA). LightCycler FastStart DNA Master SYBR green I (Roche, USA) was used for amplification in a total volume of 20 μl. According to Ct value, we calculated the sample cDNA copy number by using the standard curves, and then analyzed PCR results by ABI7000 software (Applied Biosystems, Foster City, CA, USA). Gene-specific primers for CTHRC1, CD133, CD44, N-cadherin, vimentin, E-cadherin and β-actin were designed using the Primer Premier software (Premier Biosoft International, Palo Alto, CA) as listed in Table 5. β-actin was used as an internal reference.

**Table 5:** Multivariate analysis of Disease-free survival in EOC patients

	B	Hazard ratio	95.0% CI for Exp (B)		*p*-value
			Lower	Upper	
Stage (I/II vs. III/IV)	1.262	3.53	1.16	10.75	0.026
Tumor grade (I vs. II-III)	1.098	3.00	1.07	8.38	0.036
CTHRC1 expression (high vs. low	1.121	3.07	1.45	6.48	0.003

### Western blotting and immunofluorescence staining

Western blotting was performed as previously described [12]. The antibodies for CTHRC1 (Abcam, Ltd., USA), β-catenin, GAPDH and LaminB1 (Abcam, Cambridge, UK) in 5% milk/TBST (tris-buffered saline Tween-20) were used. The signal was observed by using an enhanced chemiluminescence (ECL) plus kit (Milipore). The relative expression of each protein was determined using NIH Image J software. As for the immunofluorescence assay, cells cultured in the chamber slides were probed with CTHRC1 and β-catenin according to the manufacturer's instructions. The florescein isothiocyanate (FITC)-coupled and Rhodamine (Rhod)-coupled secondary antibodies were purchased from Molecular Probes. Treated cells were observed and pictured through an Olympus BX51 fluorescence microscope (Olympus, Tokyo, Japan).

### Cell adhesion assay

Cell adhesion experiments were applied to test the adhesive capacity of OVCAR-3 and SKOV-3 cells. Firstly, the 96-well plates were coated with 10 μg/mL fibronectin in PBS for 2 h at 37°C, and then blocked with 1% bovine serum albumin (BSA). Centrifugation and suspension (using non-serum RPMI 1,640 with 0.1% BSA and 0.1% glucose) were subsequently performed to collect cells. In triplicate, 100 μl of 75 × 10^4^ cells/ml were plated and incubated for indicated periods (24 h, 48 h and 72 h, respectively) at 37°C. PBS was used to remove non-adherent cells by washing carefully. The adherent cells were examined under an IX71 inverted microscope (Olympus Corp, Tokyo, Japan).

### MTT cell viability assay

Cells were incubated in 96-well plates at a density of 1 × 10^4^ cells per well for 24 h. Then, at different time points, MTT reagent (3-[4,5-dimethylthiazol-2-yl]-2,5-diphenyl tetrazolium bromide) (10 μl) was added to each well and incubated for another 4 h at 37°C. Then, 150μl of dimethyl sulfoxide was added to each well and mixed for 10 min. A fluorescence microplate reader (Molecular Devices) was applied to measure the spectrometric absorbance of 550 nm. Each sample had three independent replicates.

### Soft agar colony formation

Anchorage-independent growth was determined by seeding 3.0 × 10^4^ OVCAR-3 and SKOV-3cells transfected with pcDNA3.1-CTHRC1 and siRNA-CTHRC1 in 0.33% agarose (SeaPlaque, FMC BioProducts Rockland, ME, USA) in 12-well plates. Then PDGF-BB (50 ng/ml) was added into half of the wells. After 7 days in 37°C incubator, colonies were monitored and counted under Axiovert 40 CFL microscope.

### Transwell invasion assay

To measure the invasive ability, we use 24-well BioCoat cell culture inserts (Costar, New York, NY, USA) with 8-μm-porosity polyethylene terephthalate membranes coated with Matrigel (Cultrex, MD, USA). Cells transfected with vector, pcDNA3.1-CTHRC1 or siRNA-CTHRC1 were then seeded in the upper chamber of a transwell plate (2 × 10^4^ cells/well), and the medium containing 10% FBS served as a chemoattractant in the lower chamber. After incubating for 24 h, cells that did not migrate or invade the pores were removed. The cells trapped on the bottom side of the membrane were fixed with 1% paraformaldehyde and stained with hematoxylin. IX71 inverted microscope (Olympus Corp, Tokyo, Japan) was used to count cells from ten different fields on every membrane, these counts from three independent experiments were averaged.

### Luciferase reporter assay

SKOV-3 cells treated with vector, pcDNA3.1-CTHRC1and DKK1 (R&D, USA) were seeded in 24-well plates and were transfected with 200ng β-catenin/TCF firefly luciferase reporter plasmid (TOP or FOP), and 1ng of pRL-SV40 Renilla luciferase. After 36 hours of incubation, the luciferase activities of cell lysates were measured using the Dual-Luciferase Reporter Assay System (Promega, USA) according to the manufacturer's instructions.

### *In vivo* xenograft studies in the athymic nude mice

For the xenograft assay, female nude mice (about 8 weeks of age) were anesthetized with sodium pentobarbital (50 mg/kg) in a sterile environment. Then, OVCAR3, OVCAR3/CTHRC1-siRNA, SKOV3 and SKOV3/pcDNA3.1-CTHRC1 cells (2 × 10^6^) in 50 μl of PBS were subcutaneously injected into the flank of individual nude mice using one-milliliter syringes with hypodermic needles. Different time after inoculation, the mice were killed and tumor tissues were immersed in 10% neutral buffered formalin overnight for the immunohistochemical study. For IHC staining, the primary antibodies were E-cadherin, vimentin, Snail, Slug and Twist (Abcam, USA). Enzyme-linked immunosorbent assay was used to measure the secreted CTHRC1 protein level in the serum. Tumor growth was monitored by a caliper and an IVIS Imaging System (Xenogen). Living Image and Xenogen software was used to analyze the images and bioluminescent signals. We analyzed H&E staining status and BLI images of mice to define tumor metastasis. All animal experiments were performed according to the protocols approved by the medical ethical committee of Sun Yat-sen University.

### Statistical analysis

All data analyses were conducted using SPSS 16.0 statistical software package. The Chi-square test was used to assess the correlation between CTHRC1 expression and various clinicopathological parameters. The Student's *t*-test was applied to determine the statistical significance of the observed differences between groups. Survival curves were generated by using the Kaplan-Meier method with log-rank test. Univariate and multivariate analysis was performed using the Cox proportional hazard models to assess the prognostic factors for survival time. ROC curve analysis was conducted to determine the cutoff point of high or low CTHRC1 level. A *p*-value < 0.05 was considered to be significantly different.

## SUPPLEMENTARY TABLE


