# Correction: High expression of CTHRC1 promotes EMT of epithelial ovarian cancer (EOC) and is associated with poor prognosis

**DOI:** 10.18632/oncotarget.27370

**Published:** 2020-02-25

**Authors:** Minzhi Hou, Zhiqiang Cheng, Hongwei Shen, Shanyang He, Yang Li, Yunping Pan, Chongjin Feng, Xinlin Chen, Yang Zhang, Millicent Lin, Liantang Wang, Zunfu Ke

**Affiliations:** ^1^ Department of Pathology, the First Affiliated Hospital, Sun Yat-sen University, Guangzhou, Province Guangdong, P.R. China; ^2^ Department of Pathology, ShenZhen People’s Hospital, Second Clinical Medical College of Jinan University, Shenzhen, Guangdong, P.R. China; ^3^ Department of Gynecology, the First Affiliated Hospital, Sun Yat-sen University, Guangzhou, Province Guangdong, P.R. China; ^4^ Department of Stomatology, the First Affiliated Hospital, Sun Yat-sen University, Guangzhou, Province Guangdong, P.R. China; ^5^ Department of Preventive Medicine and Biostatistics, School of Basic Medical Science, Guangzhou University of Chinese Medicine, Guangzhou, P.R. China; ^6^ Biomedical Engineering, University of Texas at El Paso, El Paso, Texas, USA; ^7^ Department of Molecular and Medical Pharmacology, Crump Institute for Molecular Imaging (CIMI), California NanoSystems Institute (CNSI), University of California, Los Angeles, California, USA

**This article has been corrected:** Due to errors during figure assembly, the Figure 8A control image is incorrect. The corrected Figure 8 is shown below. In addition, in the sections RESULTS and MATERIALS AND METHODS, the terms “nude mice” or “athymic nude mice” are incorrect; they have been changed in the paragraphs below to 'NOD.Cg-PrkdcscidIl2rgtm1Sug/JicCrl (NOG) mice'. The authors declare that these corrections do not change the results or conclusions of this paper.

Original article: Oncotarget. 2015; 6:35813–35829. 35813-35829. https://doi.org/10.18632/oncotarget.5358

## *In vivo* xenograft studies in the NOD.Cg-PrkdcscidIl2rgtm1Sug/JicCrl (NOG) mice

For the xenograft assay, female NOD.Cg-PrkdcscidIl2rgtm1Sug/JicCrl (NOG) mice (about 8 weeks of age) were anesthetized with sodium pentobarbital (50 mg/kg) in a sterile environment. Then, OVCAR3, OVCAR3/CTHRC1-siRNA, SKOV3 and SKOV3/pcDNA3.1-CTHRC1 cells (2 × 10^6^) in 50 μl of PBS were subcutaneously injected into the flank of individual NOD.Cg-PrkdcscidIl2rgtm1Sug/JicCrl (NOG) mice using one-milliliter syringes with hypodermic needles. Different time after inoculation, the mice were killed and tumor tissues were immersed in 10% neutral buffered formalin overnight for the immunohistochemical study. For IHC staining, the primary antibodies were E-cadherin, vimentin, Snail, Slug and Twist (Abcam, USA). Enzyme-linked immunosorbent assay was used to measure the secreted CTHRC1 protein level in the serum. Tumor growth was monitored by a caliper and an IVIS Imaging System (Xenogen). Living Image and Xenogen software was used to analyze the images and bioluminescent signals. We analyzed H&E staining status and BLI images of mice to define tumor metastasis. All animal experiments were performed according to the protocols approved by the medical ethical committee of Sun Yat-sen University.

## CTHRC1 is closely associated with EMT status in xenograft models

To reveal the role of CTHRC1 in the metastasis and EMT of EOC cells, we explored the metastatic activity of OVCAR3/CTHRC1-siRNA and SKOV3/pcDNA3.1-CTHRC1 in NOD.Cg-PrkdcscidIl2rgtm1Sug/JicCrl (NOG) mice. We found that OVCAR3/CTHRC1-siRNA cells predominantly localized to tumor nodules in the primary injection sites compared to control. However, SKOV3/pcDNA3.1-CTHRC1 cells formed multiple tumors in the peritoneum cavity. The number of metastatic nodules was measured according to the fluorescence signal and H&E staining. As shown in Figure 8A and 8B, SKOV3/pcDNA3.1-CTHRC1 cells formed a greater number of metastases than SKOV3 cells in abdomen (3.00 ± 1.05 vs. 1.30 ± 0.48, *p* < 0.01, respectively). These *in vivo* results confirmed the role of CTHRC1 in the promotion of EOC invasion.

**Figure 8:**
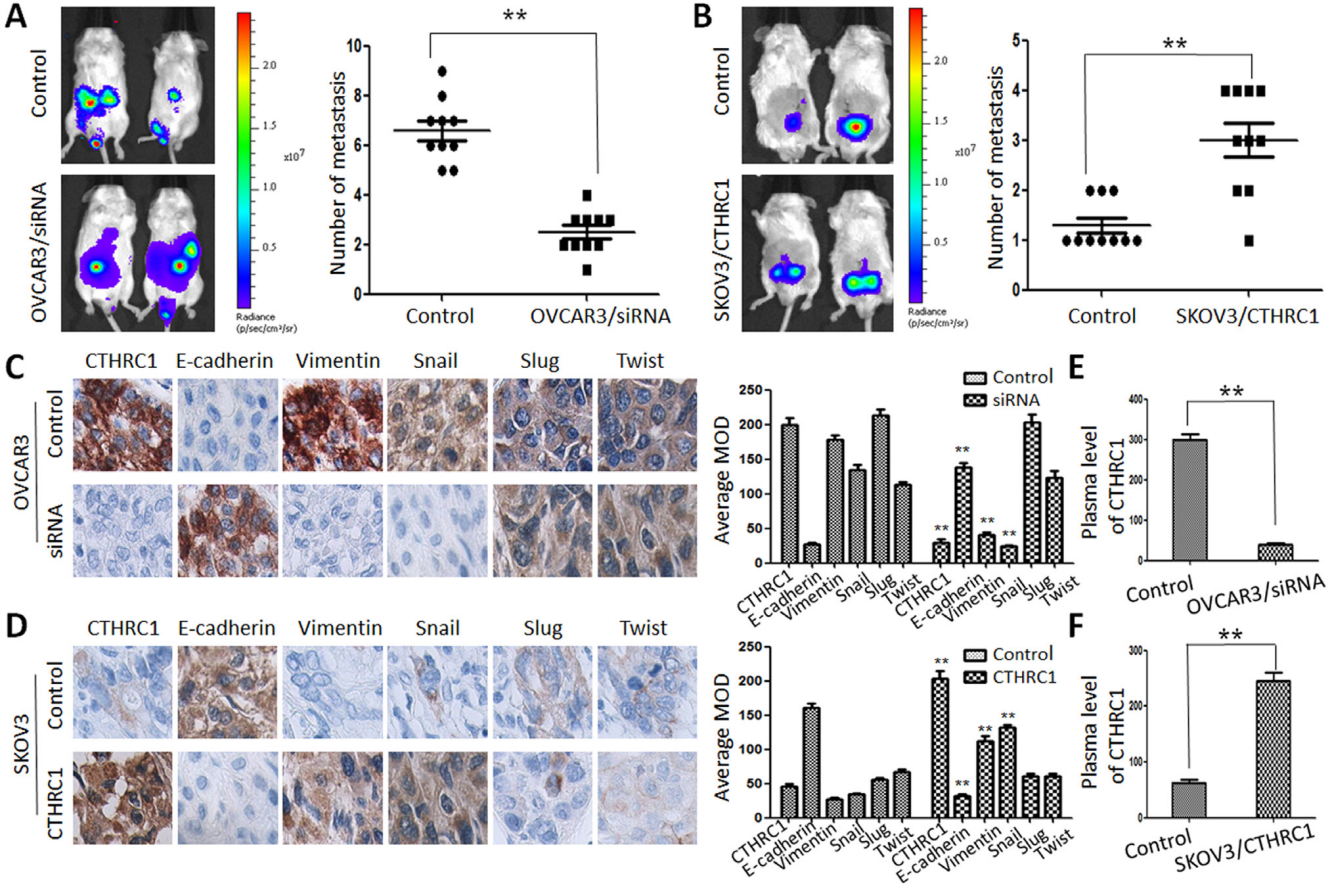
CTHRC1 promoted tumor metastasis by regulating EMT *in vivo*.

(**A**, **B**) Representative BLI images of mice bearing OVCAR3/CTHRC1-siRNA or SKOV3/pcDNA3.1-CTHRC1 tumors with metastatic lesions. Mice (*n* = 10) were imaged six weeks later to observe local tumor growth and metastasis (**p < 0.01). (**C**, **D**) CTHRC1 promoted the EMT process *in vivo*. IHC demonstrated that CTHRC1 increased vimentin and Snail expression, and decreased E-cadherin expression in the primary xenograft tumor tissues (magnification: 200 ×). (**E**, **F**) In the mice with OVCAR3/CTHRC1-siRNA xenograft tumor, the CTHRC1 protein levels in the plasma were lower than that in the control. In contrast, in the SKOV3/pcDNA3.1-CTHRC1 group, the CTHRC1 protein levels in the plasma were higher than that in the control (**p < 0.01).

