# Supplementary material for: High expression of CTHRC1 promotes EMT of epithelial ovarian cancer (EOC) and is associated with poor prognosis
Source: Oncotarget. 2015 Oct 3;6(34):35813–29. doi: 10.18632/oncotarget.5358 (PMC4742143; doi:10.18632/oncotarget.5358)
Supplement: Supplementary file 1 [file oncotarget-06-35813-s001.pdf]

SUPPLEMENTARY TABLE

Supplementary Table S1: Relative protein expression levels of CTHRC1 and nuclear β-catenin in 88 EOC tissue samples were tested by Western blotting

Number	CTHRC1		nuclear β-catenin	
	Mean	SD	Mean	SD
1	4.3	0.43	3.8	0.26
2	2.7	0.15	1.8	0.13
3	3.6	0.26	2	0.21
4	1.1	0.09	0.3	0.02
5	2.4	0.12	1.7	0.2
6	2.5	0.23	1.8	0.1
7	3	0.35	2.2	0.21
8	5.9	0.44	4.3	0.33
9	2.7	0.34	2.6	0.23
10	2.5	0.22	2.1	0.1
11	4.9	0.33	3.9	0.1
12	4.3	0.32	3.7	0.1
13	1.1	0.08	0.8	0.03
14	2.7	0.31	2.1	0.2
15	4.3	0.57	3.9	0.23
16	3.7	0.33	2.4	0.23
17	3.5	0.29	2.3	0.21
18	7.1	0.57	5.2	0.35
19	4.6	0.36	2.9	0.28
20	4.3	0.33	3.7	0.31
21	4.4	0.41	3.8	0.24
22	5.1	0.12	4.1	0.26
23	1.7	0.03	1.6	0.06
24	2.1	0.31	1.2	0.09
25	3.1	0.23	1.9	0.13
26	3.2	0.15	1.97	0.14
27	3.7	0.36	2.32	0.22
28	4.2	0.22	3.12	0.31
29	5.1	0.32	3.97	0.26
30	0.9	0.06	0.24	0.01
31	3.3	0.21	2.32	0.11
32	4.2	0.29	3.57	0.23

(*Continued*)

Number	CTHRC1		nuclear β-catenin	
	Mean	SD	Mean	SD
33	2	0.13	1.35	0.13
34	1	0.05	0.51	0.05
35	2	0.16	2.33	0.23
36	3.2	0.31	2.43	0.19
37	5.33	0.19	4.09	0.19
38	4.97	0.33	3.78	0.25
39	6.12	0.48	5.23	0.33
40	7.12	0.64	5.32	0.29
41	3.45	0.26	1.09	0.08
42	4.44	0.31	5.12	0.56
43	4.32	0.23	3.59	0.31
44	4.21	0.23	4.01	0.11
45	4.78	0.64	3.99	0.12
46	5.09	0.24	4.35	0.39
47	5.32	0.36	4.54	0.44
48	1.98	0.51	1.09	0.06
49	1.88	0.39	0.98	0.03
50	2.89	0.25	0.91	0.07
51	3.34	0.31	2.56	0.12
52	5.21	0.47	4.01	0.12
53	4.55	0.26	5.34	0.13
54	4.67	0.41	4.01	0.12
55	3.78	0.31	3.21	0.12
56	3.21	0.28	2.87	0.14
57	2.76	0.24	2.21	0.23
58	2.67	0.21	1.97	0.11
59	2.99	0.29	0.98	0.11
60	2.98	0.33	2.31	0.12
61	2.71	0.34	2.34	0.1
62	2.56	0.26	3.21	0.13
63	1.22	0.25	0.67	0.03
64	1.11	0.13	0.32	0.01
65	3.89	0.41	3.49	0.23
66	1.45	0.12	0.98	0.12
67	5.44	0.22	2.1	0.21
68	5.89	0.32	4.98	0.33

(*Continued*)

Number	CTHRC1		nuclear β-catenin	
	Mean	SD	Mean	SD
69	3.29	0.21	3.01	0.23
70	4.11	0.36	3.89	0.26
71	4.09	0.22	3.32	0.34
72	3.09	0.21	2.23	0.36
73	3.43	0.31	2.91	0.38
74	3.32	0.33	3.01	0.26
75	2.56	0.21	1.92	0.11
76	2.71	0.31	2.23	0.13
77	2.98	0.26	1.99	0.23
78	3.08	0.41	2.97	0.21
79	4.01	0.31	4.45	0.31
80	4.9	0.28	1.78	0.11
81	0.78	0.34	0.65	0.06
82	1.98	0.29	2.12	0.16
83	3.32	0.37	3.21	0.36
84	4.77	0.26	3.98	0.43
85	3.91	0.28	3.11	0.29
86	3.21	0.31	2.89	0.22
87	2.69	0.18	2.96	0.23
88	2.77	0.17	1.86	0.35

CTHRC1 and nuclear β-catenin expression levels were normalized against GAPDH protein.
